# The Leprosy Case

**Published:** 1916-05-27

**Authors:** 


### The Hospital

The Workers' Newspaper of Administrative Medicine and Institutional Life, Administration, National Insurance and Health.

No. 1563, Vol. LX. SATURDAY, MAY 27, 1916. PRICE ONE PENNY.

### THE LEPROSY CASE.

Whether leprosy is or is not a contagious disease has been a question in many discussions, and over it much controversial ink and argumentative eloquence have been spent. A prompt and confident answer has recently issued from the jury-box, and whatever the scientific or the legal experts may think of the value of this reply, there can be little doubt that it will be approved by the public. Rightly or wrongly, there is a traditional horror and widespread fear of the disease, and these are little likely to be relieved by medical or other assurances. Hence when a lodging-house keeper into whose house has been introduced without full disclosure a person suffering from leprosy asks a jury of his countrymen for damages, it is hardly surprising that the request receives an affirmative response. The conclusion may be subjected to technical criticism, and may possibly be ruled out as destitute of legal value, but it will stand as a popular verdict. Dermatological experts and judges of the King's Bench Division neither keep lodging-houses nor do they live in lodging-houses. They may therefore emphasise academic doctrines about leprosy and may discuss the issue of the contagiousness or non-contagiousness of the disease with literary grace and detached dexterity. But more humble mortals, who, personally or as representative of their fellows, feel the pressure of the situation, may be excused for a resolve to deal with the question in a strictly practical spirit. This, it seems to us, is the meaning of the verdict in the recent trial, and whatever may be the interpretation ultimately placed on it by the legal tribunals, it has a note of practical justice which will not easily be challenged in the general judgment. If it is not the law that the introduction of a leper into the house of a British citizen can only be accomplished with the knowledge and consent of the householder, then most people, we fancy, will agree that it is high time the law was altered.

The principal medical interest in the recent trial was, of course, the possibility of the spread of the disease by contagion. The experts called on the one side and the other agreed that there is little confident knowledge of the way in which leprosy is spread, though the majority affirmed the general view that the infection does or can pass from person to person. None of them went so far as to say that such extension is easily accomplished, but though they varied in their estimate of the degree of the risk, only one of them absolutely excluded it. In this connection it is not an unfair thing to remark that substantially there has been no opportunity for doctors in this country to acquire any considerable body of experience on this question, and in these circumstances personal views and conclusions can hardly carry a full degree of conviction and comfort to the public. The natural and reasonable determination is therefore to be on the safe side, and though such an experience is little likely to recur, we should not be at all surprised to find a demand for the inclusion of leprosy among the notifiable diseases. Nor do we see how such a demand could be resisted, though panic legislation, we confess, is little to our taste.

If we may judge from the Press reports, it seems to us that the case for contagion might be more strongly put than it was at the recent trial. Possibly a popular view to this effect may not have convincing value, but such a view is a strong one, and it exists in all countries where the disease is known. In medical circles in this country interest in the question has been mainly kept alive by the crusading zeal of the late Sir Jonathan Hutchinson. He, as is well known, was the most prominent champion of the non-contagion doctrine, and he travelled far and wide to collect facts in favour of his contention that the disease is spread by the eating of salted or preserved fish. Yet he made few or no disciples, and after his visit to South Africa he allowed that "there is good reason to believe that in the Kaffir kraals of Natal the disease is communicated from person to person, especially to young children." Though holding firmly to his salt-fish hypothesis as defining the usual method of spread, he thought it possible that occasionally food contaminated by secretions direct from a leper's hand might convey the disease. Considering the source from which these qualifications come, they must have very special weight in reference to the practical handling of leprosy in any community where the disease exists or where there is any risk of its introduction. There are still stronger indications in the same direction, and this from medical authorities who have had to deal with the disease on the large scale. One of the most important of these is certainly the late Dr. Hansen, whose extensive studies of leprosy in Norway are universally recognised as authoritative in the highest possible degree, and to whose credit stands the discovery of the bacillus which is now allowed to be the cause of the disease. Dr. Hansen had the courage of his convictions, and he defended without hesitation the compulsory segregation of lepers as this is enforced by Norwegian law, imperfectly in 1856, and completely in 1885. In a discussion in London in 1902 he stated that in 1856 there were probably 2,870 lepers in Norway, while at the date on which he spoke the numbers did not exceed 500. He fully admitted that in many circumstances the bacillus seemed to have little capacity to spread from person to person, and that personal cleanliness was a powerful preventive measure, but equally he had no doubt that the elimination of the disease in Norway was due to the measures taken to diminish the opportunities of spread by contagion. "We aim," he said, "at prevention, and the one measure in which I have faith is isolation." It may be quite true that existing conditions in this country make the chance of any extension of the disease an extremely remote one and experience to date seems to establish this view, for though lepers certainly come here, no one has been able to quote the occurrence of new cases. Broadly, it may be said that until some one can prove an alternative method, the case for contagion holds the field, and in these circumstances most people will agree that exposure to the risk—even allowing that this is but slight—ought not to be inflicted without warning. This may be taken as the view adopted by the jury in the recent case, and with it we are by no means disposed to quarrel.

